# A Sensitive Yellow Fever Virus Entry Reporter Identifies Valosin-Containing Protein (VCP/p97) as an Essential Host Factor for Flavivirus Uncoating

**DOI:** 10.1128/mBio.00467-20

**Published:** 2020-04-14

**Authors:** Harish N. Ramanathan, Shuo Zhang, Florian Douam, Katrina B. Mar, Jinhong Chang, Priscilla L. Yang, John W. Schoggins, Alexander Ploss, Brett D. Lindenbach

**Affiliations:** a Department of Microbial Pathogenesis, Yale University, New Haven, Connecticut, USA; b Department of Molecular Biology, Princeton University, Princeton, New Jersey, USA; c Department of Experimental Therapeutics, The Baruch S. Blumberg Institute, Doylestown, Pennsylvania, USA; d Department of Microbiology, University of Texas Southwestern Medical Center, Dallas, Texas, USA; e Department of Microbiology and the Blavatnik Institute, Harvard Medical School, Boston, Massachusetts, USA; University of Pittsburgh School of Medicine

**Keywords:** flavivirus, nucleocapsid, uncoating, viral entry

## Abstract

While the basic mechanisms of flavivirus entry and fusion are understood, little is known about the postfusion events that precede RNA replication, such as nucleocapsid disassembly. We describe here a sensitive, conditionally replication-defective yellow fever virus (YFV) entry reporter, YFVΔSK/Nluc, to quantitively monitor the translation of incoming, virus particle-delivered genomes. We validated that YFVΔSK/Nluc gene expression can be neutralized by YFV-specific antisera and requires known flavivirus entry pathways and cellular factors, including clathrin- and dynamin-mediated endocytosis, endosomal acidification, YFV E glycoprotein-mediated fusion, and cellular LY6E and RPLP1 expression. The initial round of YFV translation was shown to require cellular ubiquitylation, consistent with recent findings that dengue virus capsid protein must be ubiquitylated in order for nucleocapsid uncoating to occur. Importantly, translation of incoming YFV genomes also required valosin-containing protein (VCP)/p97, a cellular ATPase that unfolds and extracts ubiquitylated client proteins from large complexes. RNA transfection and washout experiments showed that VCP/p97 functions at a postfusion, pretranslation step in YFV entry. Finally, VCP/p97 activity was required by other flaviviruses in mammalian cells and by YFV in mosquito cells. Together, these data support a critical role for VCP/p97 in the disassembly of incoming flavivirus nucleocapsids during a postfusion step in virus entry.

## Abstract (precis)

Flaviviruses are an important group of RNA viruses that cause significant human disease. The mechanisms by which flavivirus nucleocapsids are disassembled during virus entry remain unclear. Here, we used a yellow fever virus entry reporter, which expresses a sensitive reporter enzyme but does not replicate, to show that nucleocapsid disassembly requires the cellular protein-disaggregating enzyme valosin-containing protein, also known as p97.

## INTRODUCTION

Flaviviruses are a large group of positive-strand RNA viruses classified as a genus, *Flavivirus*, within the family *Flaviviridae*, including several medically important, arthropod-borne human pathogens such as dengue virus (DENV), Japanese encephalitis virus, West Nile virus (WNV), yellow fever virus (YFV), and Zika virus (ZIKV) (1). Infectious virions are small (∼50 nm diameter), lipid-enveloped particles that display 180 copies of the envelope (E) glycoprotein and a small transmembrane protein, M, on their surfaces, surrounding an inner nucleocapsid composed of the viral capsid protein and an unsegmented RNA genome of ∼11 kb (2, 3). While the surface of flavivirus particles is relatively well-defined, nucleocapsid symmetry has been difficult to discern by cryo-electron microscopy (cryo-EM) (2, 4). Recent single-particle cryo-EM analysis and icosahedral averaging suggest that Zika virus nucleocapsid is composed of 20 trimers of dimeric capsid protein, loosely held together under the envelope by lateral interactions involving the capsid α5 helix (5). This is surprising, since α5 serves as a signal peptide for translocation of prM into the endoplasmic reticulum (ER) and must be proteolytically removed by the viral NS2B-3 serine protease in order for prM signal peptidase cleavage to occur, which is required for virus assembly (6–8). These results suggest that additional capsid protein dimers lacking α5 helix may be buried within the RNA core.

**FIG 1:**
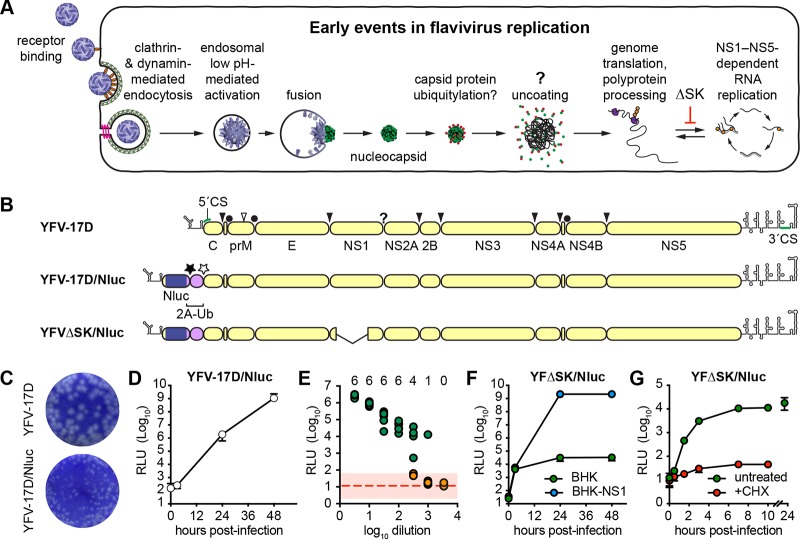
The Nluc reporter virus is a sensitive tool to monitor early events of flavivirus infection. (A) Early steps in the flavivirus life cycle, showing entry, uncoating, translation, and RNA replication. (B) Genome representation and polyprotein processing of YFV-17D, YFV-17D/Nluc, and YFVΔSK/Nluc reporter viruses used in this study. Circles represent signal peptidase cleavage sites within the YFV polyprotein; filled arrowheads represent YFV NS2B-3 serine protease cleavage sites; the open arrowhead represents a furin cleavage site; the question mark represents cleavage site by an unidentified cellular protease; filled and open stars represent the FMDV 2A translational skipping site and ubiquitin C-terminal hydrolase cleavage site, respectively. (C) Representative wells from YFV-17D and YFV-17D/Nluc plaque assays developed over 5 days with a 0.3% Avicel CL-611 overlay. (D) Time course of Nluc expression after YFV-17D/Nluc infection (MOI of 0.3) of BHK-21 cells. These results are representative of five experiments conducted over different transfection conditions and time scales. (E) Results of a 24-h endpoint dilution assay performed in 6-fold replicate. Wells were scored as positive (green) or negative (orange) based on whether they were >2σ (pink rectangle) away from the mean of uninfected controls (dotted red line); the numbers at the top of the graph represent the number of positive wells at each dilution. This experiment was performed three times with similar results. (F) Representative time course of Nluc expression after YFV-ΔSK/Nluc infection (MOI of 0.1) of BHK-21 or BHK-NS1 cells. This experiment was performed three times, each with three technical replicates. (G) Representative time course of Nluc expression after YFV-ΔSK/Nluc infection (MOI of 0.1) of untreated or CHX-treated BHK-21 cells. This experiment was performed three times, each with three technical replicates. Error bars represent standard deviations from the mean.

Flavivirus infection initiates through interaction of the viral E glycoprotein with one or more host cell attachment factors that serve to concentrate the virions on cell surface, as well as virus entry receptors that have only been partially identified (9). Virus internalization occurs through clathrin- and dynamin-dependent receptor-mediated endocytosis (10). As internalized virus particles pass through endosomes they encounter low pH, which triggers rearrangement of the E glycoprotein, leading to fusion and release of viral nucleocapsids into the cytoplasm of infected cells (Fig. 1A).

Once in the cytoplasm, flavivirus nucleocapsids are presumably disassembled to release the viral genome, which must be translated in order to initiate replication. The viral genome encodes a single open reading frame, which is translated to produce a polyprotein that is processed by viral and cellular proteases to yield three structural proteins—capsid (C), pre-M (prM), and E—as well as seven nonstructural (NS) proteins: NS1, NS2A, NS2B, NS3, NS4A, NS4B, and NS5 (Fig. 1B). NS1 is a secreted glycoprotein that is required for RNA replication; it forms dimers that remain peripherally associated with the inner leaflet of the ER membrane or on the cell surface, as well as secreted hexameric lipoprotein particles that induce strong humoral responses and contribute to flavivirus pathogenesis (3, 11). NS2A, NS2B, NS4A, and NS4B are polytopic membrane proteins required for RNA replication. NS2B and NS3 form a membrane-anchored enzyme complex with serine protease and RNA helicase activities essential for viral polyprotein processing and RNA replication (3, 12). NS5 is the viral RNA-dependent RNA polymerase and RNA capping enzyme (3, 13, 14). Once translated, the NS proteins presumably recruit the viral genome out of translation and into an RNA replication complex.

Little is known about the process of flavivirus nucleocapsid disassembly. Nucleocapsids obtained by solubilizing WNV particles with nonionic detergent are partially accessible for translation (i.e., a subset of viral proteins can be translated in *in vitro* translation reactions), suggesting that nucleocapsids may spontaneously uncoat (15). On the other hand, intact nucleocapsids can be isolated from detergent-solubilized tick-borne encephalitis virus particles (16); these nucleocapsids dissociate in high salt (0.5 M sodium chloride). In cell culture, however, DENV capsid protein must be ubiquitylated in order for nucleocapsid uncoating and genome translation to occur (17), suggesting that uncoating is an active process *in vivo*.

Here, we describe a sensitive, conditionally replication-defective YFV reporter virus designed to probe the early events of the flavivirus life cycle. We validate the specificity of this reporter to monitor YFV entry and prereplication events, confirm that YFV entry requires ubiquitylation, and then used this tool to examine the hypothesis that YFV nucleocapsids are disassembled by valosin-containing protein (VCP), also known as p97.

VCP/p97 is a conserved and abundant eukaryotic AAA+ ATPase that uses the energy released by ATP hydrolysis to unfold ubiquitylated proteins and extract them from large macromolecular complexes (18, 19). VCP/p97 forms hexameric double-ring structures with a central pore (20, 21); each subunit contains an N-terminal regulatory domain and two RecA-like ATPase domains. VCP/p97 plays an essential role in protein homeostasis and genome stability. It is therefore an attractive target for anticancer therapies and several potent and specific VCP/p97 inhibitors have been developed (22). We show here that VCP/p97 is required for a postfusion, prereplication event during YFV entry.

## RESULTS

### YFV reporter viruses are sensitive tools to monitor early events of flavivirus infection.

A key challenge to studying the early life cycle events of flaviviruses is in detecting the translation of incoming, virion-released genomes. While viruses can be engineered to express sensitive reporter genes, signals produced by translation of the incoming viral genome are soon overwhelmed by translation of genomes produced by RNA replication. Therefore, in order to specifically study early, prereplication events in the life cycle of flaviviruses, we sought to uncouple translation from RNA replication by constructing a YFV strain 17D (YFV-17D) reporter that is conditionally defective for RNA replication. The YFV-17D mutant YFVΔSK, which contains a large, in-frame deletion within the essential NS1 gene, is incapable of initiating RNA replication but can be complemented in *trans* (23). Thus, in the absence of NS1, a YFVΔSK-based reporter virus should allow viral entry, fusion, uncoating, and primary translation of the incoming genome to be monitored (Fig. 1A).

First, we constructed a full-length, infectious YFV-17D reporter virus that expresses the nanoluciferase (Nluc) enzyme, based on previously described flavivirus reporter designs (24–26). We chose Nluc because of its smaller size (19.1 kDa; 171 codons), enhanced stability, and exquisite sensitivity compared to other luciferases (27). A cassette encoding Nluc, the foot-and-mouth disease virus 2A translational-skipping peptide (NFDLLKLAGDVESNPG–P; where “–” signifies the unformed peptide bond), and a ubiquitin monomer (MQIFV…LRGG|; where “|” signifies cleavage by a ubiquitin C-terminal hydrolase) was inserted in frame into the YFV-17D infectious clone after the first 25 codons of the YFV-17D C gene, which contains the essential 5′ RNA cyclization sequence (28, 29), followed by the entire YFV polyprotein coding sequence, to generate YFV17D/Nluc (Fig. 1B). After transfection into BHK-21 cells, YFV-17D/Nluc RNA transcripts replicated and gave rise to infectious virus with peak titers similar to wild-type YFV-17D (≥1 × 10^7^ PFU/ml at 48 h posttransfection) but had a small plaque phenotype (Fig. 1C). Similar replication impairments have been reported with other flavivirus reporter constructs (26). Nluc expression was stably maintained for at least three serial virus passages in BHK-21 cells; we did not specifically address the long-term stability of the Nluc insert. Based on prior reports of flavivirus insert instability, we expect that Nluc expression will be lost with passage and therefore limited our experiments to early passage virus stocks. Importantly, YFV-17D/Nluc was able to infect and replicate in BHK cells, as observed by the robust accumulation of Nluc activity over time (Fig. 1D). Robust Nluc expression was also observed upon YFV-17D/Nluc infection of other established cell lines, including HEK 293, HeLa, Huh-7.5, SW-13, and primary mouse fibroblasts (data not shown). Furthermore, Nluc expression levels directly correlated with the amount of input virus in an endpoint dilution assay (Fig. 1E); notably, some replicates became Nluc-negative at higher dilutions, indicating that an endpoint had been reached (i.e., some replicate wells received no virus, while other wells received one or a few viruses). Nluc activity was 10- to 100-fold higher in positive wells around the endpoint, which likely received only a single virus particle, than negative wells (Fig. 1E). Based on this, we were able to calculate a 50% tissue culture infectious dose (TCID_50_) of 3.54 × 10^4^ TCID_50_/ml, which was similar to the plaque infectivity titers (3.25 × 10^4^ PFU/ml) of this same early-harvest (18 h posttransfection), low-titer stock. We consistently noted that virus stocks harvested after cytopathic effects became evident contained significant Nluc activity, presumably due to enzyme release into the conditioned medium by cell lysis. Thus, early virus harvests provided optimal signal/noise ratio without requiring extensive washing during virus inoculation (see Materials and Methods for detailed information). Taken together, these data show that YFV-17D/Nluc is a sensitive reporter virus for measuring YFV-17D replication and gene expression at both early and late times postinfection.

Next, we generated a conditionally replication-defective construct, YFVΔSK/Nluc (Fig. 1B), containing a large in-frame deletion within the essential NS1 gene (23). Upon transfection of RNA transcripts into BHK-21 cells that express YFV NS1 (BHK-NS1), YFVΔSK/Nluc replicated, expressed Nluc, and produced infectious virus. YFVΔSK/Nluc virus infected BHK-NS1 cells and expressed robust Nluc activity (Fig. 1F, blue circles); however, infection of parental BHK-21 cells, which do not express NS1, led to modest but significant levels of Nluc expression (Fig. 1F, green circles). To confirm that the Nluc activity observed in BHK-21 cells that lack NS1 expression was due to the entry and translation of YFVΔSK/Nluc, we performed an additional time course in the presence or absence of cycloheximide (CHX), a potent inhibitor of translation. Nluc expression was detectable as early as 30 min postinfection, increased >10-fold higher than in CHX-treated cells by 90 min postinfection, plateaued by 7 h postinfection, and that Nluc activity persisted for 24 h postinfection (Fig. 1G). Given the tight requirement for NS1 in flavivirus RNA replication, these results demonstrate that YFVΔSK/Nluc is a sensitive reporter to measure the translation of incoming viral genomes at early times postinfection.

### Nluc reporter viruses mimic authentic flavivirus infection and Nluc activity correlates with cellular entry.

**FIG 2:**
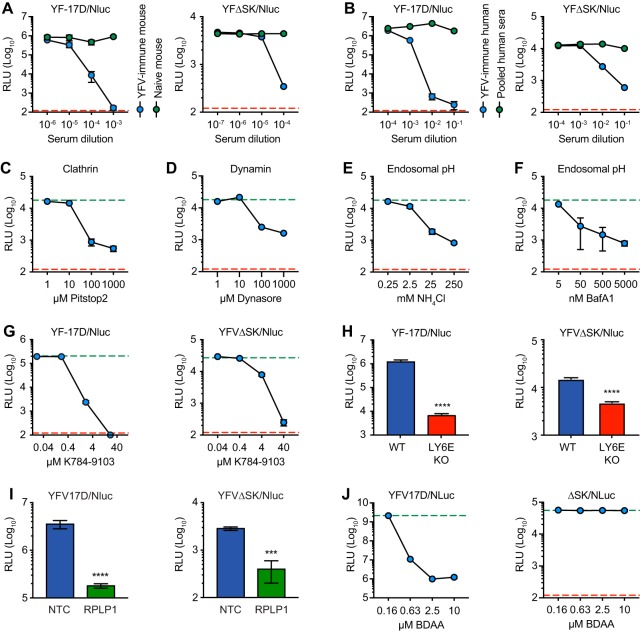
Nluc reporter viruses recapitulate authentic flavivirus infection. (A) Nluc expression after 24 h infection with YFV-17D/Nluc (MOI of 0.3; left panel) or YFVΔSK/Nluc (MOI of 0.1; right panel) pretreated with YFV-immune or control mouse sera in BHK cells. (B) Nluc expression after 24 h infection with YFV-17D/Nluc (MOI of 0.3; left panel) or YFVΔSK/Nluc (MOI of 0.1; right panel) pretreated with YFV-immune or pooled control human sera. (C) Nluc expression at 5 h postinfection with YFVΔSK/Nluc (MOI of 0.1) in cells treated with Pistop2, a potent and specific inhibitor of clathrin-mediated endocytosis. (D) Nluc expression at 5 h postinfection with YFVΔSK/Nluc (MOI of 0.1) of cells treated with Dynasore, a potent and specific inhibitor of dynamin. (E) Nluc expression from YFVΔSK/Nluc infection (MOI of 0.1) of cells treated with NH_4_Cl, which buffers the endosomal compartment. (F) Nluc expression at 5 h postinfection with YFVΔSK/Nluc (MOI of 0.1) of cells treated with Bafilomycin A, an inhibitor of endosomal acidification. (G) Nluc expression after 24 h infection with YFVΔSK/Nluc (MOI of 0.1) treated with K784-9103, a potent and specific inhibitor of flavivirus fusion. (H) Nluc expression after infection of wild-type (WT) U2OS or LY6E knockout (KO) U2OS cells with YFV/Nluc (MOI of 0.1) for 24 h (left panel) or YFVΔSK/Nluc (MOI of 0.1) for 6 h (right panel). (I) Nluc expression in HeLa cells transfected with nontargeting control (NTC) or RPLP1-specific siRNAs for 24 h and then infected with YFV/Nluc (MOI of 0.1) for 24 h (left panel) or YFVΔSK/Nluc (MOI of 0.1) for 6 h (right panel). This experiment was repeated twice, each in quadruplicate. (J) Nluc expression after 24 h infection with YFV-17D/Nluc (MOI of 0.3; left panel) or YFVΔSK/Nluc (MOI of 0.1; right panel) in cells treated with BDAA, a potent and specific inhibitor of YFV RNA replication. In all experiments, carrier control treatments are indicated by green dotted lines and the limit of Nluc detection (in parallel CHX-treated cells) by red dotted lines. All data are representative of experiments performed three times, in triplicate except for panel H, which was performed in quadruplicate. Error bars represent standard deviations from the mean. Statistical significance was calculated by using one-way analysis of variance (ANOVA; ****, *P* < 0.0001; ***, *P* < 0.001).

To further validate the utility of YFVΔSK/Nluc for monitoring early stages of virus infection, we sought to determine whether it exhibited known features of flavivirus entry and replication. First, we sought to determine whether Nluc expression was sensitive to YFV-specific neutralizing antibodies. As shown in Fig. 2A, Nluc expression from both YFV-17D/Nluc and YFVΔSK/Nluc was neutralized by serum from an IFNAR1^−/−^ mouse immunized with YFV-17D, while nonimmune control serum did not neutralize Nluc expression. Similarly, serum from a human YFV-17D vaccinee neutralized both reporter viruses, while pooled human serum did not (Fig. 2B). Together, these data confirm that Nluc expression is dependent on infectivity of the YFV reporter viruses.

Flaviviruses enter target cells via receptor-mediated endocytosis, which requires clathrin and dynamin, and delivery to endosomes, where viral fusion is induced by the low pH of this compartment (30). Consistent with this viral entry pathway, expression of Nluc by YFVΔSK/Nluc was sensitive to Pitstop2, an inhibitor of clathrin-coated pit formation (Fig. 2C), and to Dynasore, an inhibitor of dynamin (Fig. 2D). Furthermore, expression of Nluc by YFVΔSK/Nluc was sensitive to ammonium chloride (NH_4_Cl), a weak base that buffers endolysosomal compartments (Fig. 2E), and to bafilomycin A1 (BafA1), an inhibitor of the vacuolar H^+^-ATPase pump (Fig. 2F). As shown in Fig. 2G, YFV-mediated Nluc expression was sensitive to K784-9103, a small-molecule that binds to DENV E protein and inhibits membrane fusion of DENV and other flaviviruses (31). Furthermore, YFV-mediated Nluc expression was reduced by genetic ablation of LY6E (Fig. 2H), a host factor that facilitates internalization of flaviviruses and other viruses (32, 33), or by RNAi-mediated knockdown of RPLP1 (Fig. 2I), a ribosomal protein required for efficient flavivirus translation (34). These results confirm that YFV/Nluc and YFVΔSK/Nluc gene expression are dependent on clathrin, dynamin, LY6E-mediated trafficking, endosomal acidification, and YFV E-mediated fusion, consistent with the known pathways of flavivirus entry.

We also examined the effects of benzodiazepine acetic acid (BDAA), a low micromolar inhibitor of YFV RNA replication that targets NS4B (35). In contrast to the entry-specific inhibitors used above, BDAA potently inhibited Nluc expression by YFV-17D/Nluc but had no effect on Nluc expression by YFΔSK/Nluc (Fig. 2J), confirming that YFΔSK/Nluc is a sensitive reporter of prereplication events in the YFV life cycle.

### Ubiquitylation and valosin-containing protein (VCP/p97) are essential for early stages of YFV infection.

**FIG 3:**
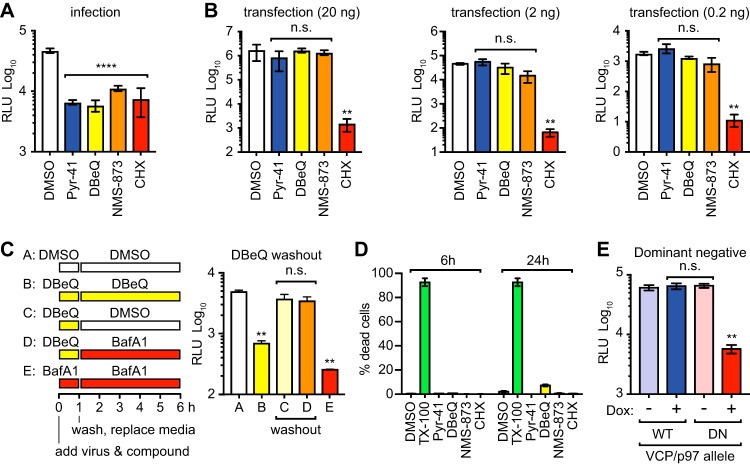
VCP/p97 is essential for an early, postfusion stage of YFVΔSK/Nluc virus infection. (A) Nluc expression at 5 h postinfection with YFVΔSK/Nluc (MOI of 0.1) of BHK cells treated with Pyr-41 (50 μM), DBeQ (10 μM), NMS-873 (300 nM), or CHX (100 μg/ml). This experiment was performed in triplicate and is representative of five independent experiments; error bars represent SD from the mean. (B) Nluc expression at 5 h posttransfection with 20 ng (left panel), 2 ng (middle panel), or 0.2 ng (right panel) YFVΔSK/Nluc RNA in BHK cells treated with the indicated compounds, as described above. This entire experiment was performed twice. (C) Nluc expression at 6 h postinfection with YFVΔSK/Nluc (MOI of 0.1) in BHK cells continuously treated with DMSO carrier control, DBeQ, or BafA1, as well as cells treated with DMSO or DBeQ and subjected to washout conditions, as indicated in the left panel and detailed in Materials and Methods. This experiment was performed three times, each in triplicate, with similar results. (D) Drug toxicity was quantified by plotting the percentage of dead cells against various inhibitor or control treatments, as above, at the indicated time points. Statistical significance was calculated by using ordinary one-way ANOVA (****, *P* < 0.0001; ***, *P* < 0.001; **, *P* < 0.01; *, *P* < 0.05; ns, not significant). This experiment was repeated twice with similar results. (E) Nluc expression at 6 h postinfection of Flp-In T-Rex-293 cells induced to express a WT or dominant negative (DN) allele of VCP/p97 for 24 h prior to infection. This experiment is representative of three independent experiments, each performed in triplicate.

Since DENV genome uncoating requires ubiquitylation, presumably of the incoming capsid protein (17), we next examined whether early YFV gene expression also requires ubiquitylation. As shown in Fig. 3A, expression of Nluc activity during YFVΔSK/Nluc infection was blocked by Pyr-41, a potent, specific, and irreversible inhibitor of the ubiquitin-activating enzyme E1 (36), confirming that ubiquitylation is required for an early, prereplication event in the YFV life cycle. To clarify the stage in entry where ubiquitylation is required, we bypassed the viral entry process by directly transfecting YFVΔSK/Nluc RNA into BHK cells. As shown in Fig. 3B, Pyr-41 did not inhibit Nluc expression after RNA transfection. These results show that ubiquitylation is required for an early step in YFV entry, upstream of genome release (i.e., uncoating), consistent with the finding that ubiquitylation is required for the disassembly of the DENV nucleocapsid (17).

These results suggested that ubiquitin can tag incoming nucleocapsids for subsequent uncoating by an unidentified host factor. The eukaryotic AAA+ ATPase VCP/p97 utilizes energy released from ATP hydrolysis to unfold ubiquitylated client proteins and extract them from larger complexes (18). We therefore tested the hypothesis that VCP/p97 promotes disassembly of the YFV nucleocapsid. As VCP/p97 is an abundant cellular protein, efficient knockdown takes several days to achieve a loss-of-function phenotype, which can cause pleiotropic effects on cells (37). Therefore, in order to specifically examine the role of VCP/p97 in YFV entry we chose two small molecules, DBeQ (an ATP competitor) and NMS-873 (an allosteric inhibitor), which work through different mechanisms of action to potently and specifically inhibit VCP/p97 in a matter of minutes, rather than days (38, 39). DBeQ and NMS-873 potently inhibited Nluc expression after infection with YFVΔSK/Nluc virus particles (Fig. 3A) but did not inhibit Nluc expression after transfection of YFVΔSK/Nluc RNA (Fig. 3B), indicating that VCP/p97 is necessary for YFV entry, prior to the delivery and translation of incoming YFV genomes.

To clarify the step at which VCP/p97 functions during YFV entry, we conducted a washout experiment. As shown in Fig. 3C, DBeQ inhibition of YFVΔSK/Nluc entry could be reversed by drug washout at 1 h postinfection. Moreover, DBeQ washout bypassed the sensitivity to BafA1, indicating that VCP/p97 functions at a postfusion step of YFV entry. We were unable to perform the converse experiment, washout of BafA1, followed by DBeQ treatment, because BafA1 washout was highly inefficient, consistent with the low nanomolar dissociation constant of this compound (40). Importantly, Pyr-41, DBeQ, NMS-873, and CHX treatments were not toxic under the concentrations and time scales used in our studies (Fig. 3D), indicating that their abilities to block viral gene expression were not simply due to cellular toxicity. Moreover, the expression of a dominant negative allele confirmed that VCP/p97 is required for early YFV gene expression (Fig. 3E). Taken together, these data suggest that VCP/p97 functions at a postfusion, prereplication step in the YFV life cycle.

### VCP/p97 is required for early events in the flavivirus life cycle.

**FIG 4:**
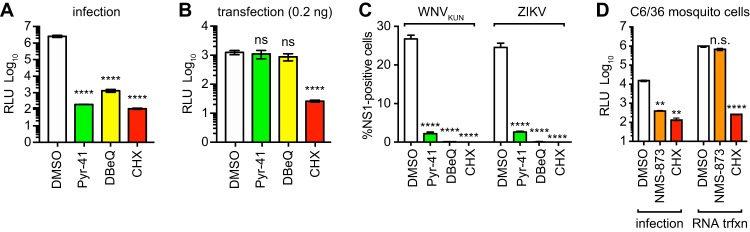
VCP/p97 activity is required for flavivirus infection. (A) Nluc expression at 18 h postinfection with YFV-17D/Nluc (MOI of 0.3) of cells treated with the indicated compounds: Pyr-41 (50 μM), DBeQ (10 μM), NMS-873 (300 nM), or CHX (100 μg/ml). (B) Nluc expression at 5 h posttransfection with 0.2 ng YFV-17D/Nluc RNA in cells treated with the indicated compounds, as described above. (C) Infection of BHK cells with WNV_KUN_ or ZIKV (each at an MOI of 1.0) in cells treated with the indicated compound, as described above. Cells were fixed at 24 h postinfection, stained for NS1 expression, and counted by flow cytometry, as detailed in Materials and Methods. (D) Nluc expression at 6 h after pretreating C6/36 mosquito cells for 1 h with the indicated compounds, followed by infection with YFΔSK/Nluc (MOI of 0.3) or transfection with 20 ng of YFΔSK/Nluc RNA (as indicated). The experiments depicted in panels A and D were performed three times, each in triplicate. The experiments depicted in panels B and C were performed twice in parallel, each in triplicate. Statistical significance was calculated by using one-way ANOVA (****, *P* < 0.0001; ***, *P* < 0.001; **, *P* < 0.01; *, *P* < 0.05; ns, not significant).

To determine whether ubiquitylation and VCP/p97 are also required for postreplication viral gene expression, we examined Nluc expression by the replication-competent YFV-17D/Nluc. As shown in Fig. 4A and B, Pyr-41 and DBeQ potently inhibited Nluc expression by 18 h postinfection with YFV-17D/Nluc, but not after 18 h posttransfection of YFV-17D/Nluc RNA. Since robust expression of Nluc activity at late times of infection (>8 h) depends on YFV-17D/Nluc replication (compare Fig. 1D, F, and G), these data suggest that ubiquitylation and VCP/p97 are specifically required for early events in the YFV life cycle.

We next examined whether other flaviviruses depend on ubiquitylation and VCP/97 activity. As shown in Fig. 4C, Pyr-41, DBeQ, and CHX all potently inhibited detectable expression of NS1 by 24 h postinfection with the Kunjin strain of WNV (WNV_KUN_) or Cambodian FSS 13025 strain of ZIKV. These data suggest that multiple flaviviruses depend on cellular ubiquitylation and VCP/p97 activity.

Finally, we examined whether VCP/p97 activity was also required for early YFV gene expression in mosquito cells. Sequence alignment showed that the Aedes albopictus and Aedes aegypti TER94 genes share 99.75% identity to one another and 84.89% identity to human VCP/p97 (see Fig. S1A to C in the supplemental material), including conserved ATPase active-site residues and residues involved in binding to allosteric inhibitors (39). Consistent with these observations, we found that NMS-873 inhibited YFVΔSK/Nluc gene expression in C6/36 Aedes albopictus cells; however, this inhibition was bypassed by transfecting YFVΔSK/Nluc RNA into these cells (Fig. 4D). These data suggest that the mosquito homolog of VCP/p97, TER94, also functions at an early step in flavivirus entry.

10.1128/mBio.00467-20.1 FIG S1 Sequence alignment of human TERA (VCP/p97) with Aedes TER94. (A) Input sequences in FASTA format. (B) CLUSTAL multiple sequence alignment by MUSCLE (v3.8); green residues indicate ATPase Walker A motif active site residues (K251 and K524 in human VCP/p97); yellow residues indicate allosteric inhibitor binding residues (K615 and N617 in human VCP/p97); “*” (asterisk) indicates identical residues; “:” (colon) indicates conserved residues that have strongly similar biochemical properties (Gonnet PAM 250 matrix scores > 0.5); “.” (period) indicates conserved residues that have weakly similar biochemical properties (Gonnet PAM 250 scores above 0 and ≤0.5). (C) Matrix of percent identity, based on the alignment. Download FIG S1, PDF file, 0.1 MB. Copyright © 2020 Ramanathan et al. 2020 Ramanathan et al. This content is distributed under the terms of the Creative Commons Attribution 4.0 International license.

## DISCUSSION

The processes of flavivirus internalization and fusion were originally characterized by using biochemical and cell biological approaches with high MOIs of radiolabeled virus particles, revealing a requirement for endocytosis and endosomal acidification (41, 42). While working with infectious, radiolabeled virus particles may be inconvenient, the use of high MOIs is potentially more problematic, since aggregates of virus particles can influence the apparent mechanisms of viral entry (43). More recently, the entry of individual, fluorescently labeled flavivirus particles has been visualized at low MOIs through live cell imaging (30, 44–46). An important caveat to this approach is that flavivirus preparations typically have relatively low specific infectivities (44, 45, 47), so it is difficult to know whether a given particle under observation is on a pathway toward productive infection.

Given these considerations, we chose to pursue a function-based approach to study the productive entry of a recombinant YFV that expresses a reporter enzyme only after viral entry and translation. Several flavivirus reporter systems have been developed, mainly for high-throughput screening of viral replication (17, 26, 48–55). In one remarkable study, Byk et al. adapted a *Renilla* luciferase-expressing DENV reporter virus to show that the incoming DENV capsid protein must be ubiquitylated prior to viral gene expression (17). However, an important consideration of this experimental design is that flavivirus-encoded reporter genes are continuously expressed, making it difficult to rigorously conclude that reporter activity was translated from an incoming viral genome versus viral RNAs produced by replication, i.e., because both viral entry and RNA replication can contribute to reporter gene expression, the translation of incoming genomes could only be inferred based on the kinetics of when reporter gene expression first became observable. Byk et al. attempted to control for this concern by using CHX to inhibit translation (17); however, CHX inhibits viral gene expression irrespective of whether the genome was delivered by a virus particle or newly synthesized by RNA replication. A further consideration is that reporter enzymes differ in their specific activities; thus, enzymes with low specific activity require higher MOIs to achieve similar sensitivity of early translation events. In this regard, it is notable that Byk et al. did not report the MOIs used in their DENV entry studies; however, several of their experiments used MOIs sufficiently high to allow incoming capsid protein to be detected by Western blotting (17). Despite these minor technical caveats, Byk et al. clearly demonstrated that incoming DENV capsid protein is degraded in a ubiquitin-dependent process and that ubiquitylation is needed before viral gene expression can be detected.

Given these considerations, we sought to build a sensitive YFV reporter specific for detecting translation of incoming viral genomes. First, we used the Nluc reporter gene, which exhibits >100-fold greater specific activity over firefly and *Renilla* luciferases (56). Second, we created a conditionally replication-defective reporter by incorporating a large, in-frame deletion in the essential NS1 gene, which can be supplied in *trans* (23, 57). The NS1 glycoprotein, which is expressed within the secretory pathway, contributes to the cytosolic process of RNA replication via interaction with the polytopic NS4A and NS4B membrane proteins, likely within the NS4A-2K-NS4B polyprotein intermediate (58–60). NS1 also has distinct membrane alteration properties (59, 61), which likely contribute to replication complex assembly. In the absence of NS1 expression, flavivirus infections are halted prior to replication complex formation and the initial round of RNA synthesis (23, 59, 62, 63). Consistent with this, robust Nluc expression by YFV-17D/Nluc was sensitive to a YFV-specific RNA replication inhibitor, BDAA, while the modest Nluc expression by YFVΔSK/Nluc was not. Thus, the YFVΔSK/Nluc virus faithfully reports on early, postfusion, prereplication events in the flavivirus life cycle.

Our experimental approach should be generally applicable to other flaviviruses. It is notable that two groups previously described NS1 deletion reporter virus systems for WNV and Omsk hemorrhagic fever virus (55, 64). These constructs were originally designed to reduce biosafety risks for high-throughput screening; our data suggest that these constructs should also be useful in dissecting early, prereplication events in the life cycle of these flaviviruses. Further improvements to our design are also possible; for instance, smaller tags, such as a split Nluc reporter (53), could improve viral titers or allow postfusion events to be monitored prior to viral genome translation.

We validated that YFVΔSK/Nluc gene expression was neutralized by YFV-specific antibodies and was dependent on several known pathways of flavivirus entry, including clathrin- and dynamin-mediated endocytosis (10, 30, 65–67), endosomal acidification (45, 66–69), E protein-dependent fusion (31), and dependence on LY6E (32, 33) and RPLP1 (34).

We then applied our YFV reporter system to address the role of ubiquitylation and protein homeostasis in flavivirus entry, which has been controversial. As part of a genome-wide RNAi screen, Krishnan et al. first reported that knockdown of ubiquitin ligase CBLL1 inhibited internalization of WNV particles into HeLa cells and that WNV entry was sensitive to proteasome inhibitors (70). However, these findings were called into question by Fernandez-Garcia et al., who found that the entry of WNV, YFV, and DENV was insensitive to rigorously validated knockdown of CBLL1 or by proteasome inhibitors (71). Furthermore, while JEV entry is also inhibited by proteasome inhibitors (72), these compounds can decrease the cellular pools of free ubiquitin (73–75), so the role of ubiquitylation versus proteasome activity in flavivirus entry has been unclear. Byk et al. brought clarity to this issue by demonstrating that ubiquitylation is required for DENV capsid disassembly (17). Furthermore, proteasome activity is dispensable for DENV entry but is responsible for the turnover of incoming capsid protein, presumably after disassembly (17).

Our studies confirm that ubiquitylation is required for flavivirus entry, although the relevant substrate(s) are unknown. Given that incoming DENV capsid protein is turned over in a proteasome-dependent manner and that inhibition of proteasome activity leads to the accumulation of a slightly higher molecular weight form of capsid protein (17), it is likely that flavivirus capsid proteins are directly ubiquitylated. It is not yet clear how nucleocapsids are targeted for ubiquitylation, nor whether there is a preferred site on capsid protein for this modification. In this regard, DENV mutants lacking lysine residues in the capsid protein were able to infect and translate their genomes normally (17), suggesting that the capsid protein may be ubiquitylated at the N terminus or other noncanonical residue(s) (76). Future work would be needed to identify of the relevant E3 ligase(s) and the type(s) of ubiquitin linkage that modify flavivirus capsid proteins.

VCP/p97 functions to unfold and extract proteins from macromolecular complexes in a ubiquitin- and ATP-dependent manner (18). For instance, VCP/p97 dissociates ubiquitylated IκBα from NF-κB, activating this transcription factor (77). VCP/p97 contributes to ER-associated degradation by extruding misfolded proteins from the secretory pathway for subsequent delivery to the proteasome (78). Similarly, VCP/p97 contributes to ribosome-associated quality control by extracting misfolded nascent polypeptides from the translation apparatus (79, 80). VCP/p97 has additional roles in extracting client substrates from chromatin, mitochondria, and other large macromolecular complexes. It is worth noting that VCP/p97 has a weak affinity for ubiquitin and relies on a large array of cofactors, which typically encode enzymatic activities to facilitate VCP/p97 substrate processing, or adaptor molecules, which simply link VCP/p97 to client substrates. Each of these adaptors and cofactors carry binding surfaces that recognize VCP/p97 and ubiquitin, respectively (81, 82). Thus, VCP/p97 contributes to diverse cellular functions based this modular cofactor- and adaptor-mediated targeting strategy.

**FIG 5:**
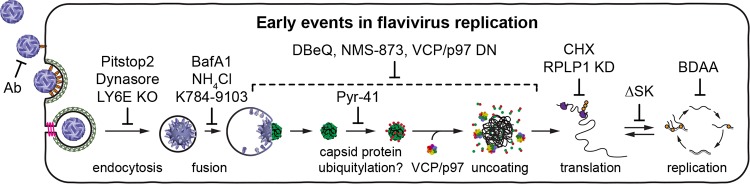
Revised model for the early, prereplication events in flavivirus infection (modified from Fig. 1A). After fusion, the nucleocapsid is exposed to the cytosol, where capsid protein (green circles) is presumably ubiquitinated (red hexagons), leading to VCP/p97-dependent nucleocapsid disassembly and release of the viral genome prior to translation. Inhibitors used in this study are indicated. The dotted line indicates the range where VCP/p97 functions in flavivirus entry.

Based on our finding that VCP/p97 activity is required for an early, postfusion event prior to the translation and replication of incoming YFV genomes, we propose a model wherein VCP/p97 functions to disassemble ubiquitylated nucleocapsids (Fig. 5). As mentioned above, direct evidence for ubiquitylation of incoming flavivirus capsid protein is currently lacking, although capsid is degraded by the proteasome in a ubiquitin-dependent manner. Although we have illustrated free nucleocapsids within the cytosol, we cannot exclude the possibility that fusion is tightly coupled to capsid ubiquitylation and disassembly, such that nucleocapsids may be ubiquitylated and disassembled as they are exposed to the cytosol. Consistent with our model, VCP/p97 activity was previously shown to be important for WNV, JEV, and DENV infection, although specific role(s) for VCP/p97 in virus entry were not determined (53, 83). Similarly, VCP/p97 is also important for an early step, upstream of N-protein degradation, during the entry of infectious bronchitis virus, a coronavirus (84). VCP/p97 also contributes to productive trafficking of NRAMP2, a cellular receptor for the entry of Sindbis virus, an alphavirus (85). Thus, VCP/p97 appears to play a general role in the entry of enveloped RNA viruses and may also contribute to RNA replication of alphaviruses (86) and hepaciviruses (87, 88). Given these findings, it will be interesting to determine whether VCP/p97 inhibitors have therapeutic potential in viral infections.

## MATERIALS AND METHODS

### Cell lines and plasmids.

Baby hamster kidney (BHK-21), clone 15 cells, BHK-21 cells stably expressing YFV-NS1 (BHK-NS1), HeLa cells, WT U2OS cells, and LY6E-knockout U2OS cells were maintained in Dulbecco minimal essential medium (DMEM; Life Technologies, Inc., Gaithersburg, MD) supplemented with 10% heat-inactivated fetal bovine serum (FBS; Omega Scientific) and 1 mM nonessential amino acids (here referred to as complete medium) at 37°C and 5% CO_2_. Flp-In T-REx-293 cells were purchased from Invitrogen and maintained at 37°C and 5% CO_2_ in complete medium containing 100 μg/ml zeocin and 15 μg/ml blasticidin. C6/36 cells were maintained at 30°C and 5% CO_2_ in MEM containing 10% FBS and 1 mM nonessential amino acids.

The construction and maintenance of pCC1/YF17D and pYF-17D/5ʹC25Venus2AUbi have been described (24, 25). Plasmid pYF17D/Nluc was constructed by replacing the Venus coding region of pYF-17D/5ʹC25Venus2AUbi with that of the Nluc gene (Promega, Madison, WI) by using standard molecular biology techniques and verified by restriction digestion and sequencing. Briefly, the SrfI–NsiI region of pYF17D/5ʹC25Venus2AUbi was subcloned into a shuttle vector, pSL1180, generating the pSL1180/S17DN intermediate. The Nluc coding region was PCR amplified with the primers YO-3008 (5′-ggg ccc GAG CTC ATG GTC TTC ACA CTC GAA GAT TTC GTT G-3′) and YO-3009 (5′-ggg ccc acc ggt CGC CAG AAT GCG TTC GCA CAG CCG CCA GC-3′) and Q5 polymerase (NEB) and then cloned into the SacI and AgeI sites of pSL1180/S17DN, resulting in replacement of Venus with Nluc. The SrfI-NsiI fragment was then subcloned back into pCC1/YF-17D to generate pYF17D/Nluc. The construction and use of pYFΔSK, as well as pSINrep21-NS1, were described previously (23, 62). To generate pYFΔSK/Nluc, the 7221-bp NsiI-ClaI fragment of pACNR/YFΔSK was subcloned into pYFV17D/Nluc cut with these same enzymes.

To generate Flp-In T-Rex-293 cells expressing WT VCP/p97 or a dominant negative mutant (K251Q/K524Q) form of VCP/97, the VCP/p97 gene was amplified from pcDNA3.1(+)/p97-WT or pcDNA3.1(+)/p97-QQ (89), kindly provided by Christian Schlieker (Yale University), by using the primers YO-3508 (5′-CCA GCC TCC GGA CTC TAG CGT TTA AAC TTA GCC ACC ATG GCT TCT GGA GCC GATT-3′) and YO-3509 (5′-TGA TGA TGA CCT GTA TGG CTA AGT ACC GAG CTC GGA TCC ACT AGT CCA GT-3′) and cloning the fragment into HindIII-cut pcDNA5/FRT/TO (Invitrogen) by using Gibson assembly (NEB). The resulting vectors were cotransfected into Flp-In T-Rex-293 cells with pOG44 (at a 1:9 mass ratio), and stable cells were selected in complete medium containing 50 μg/ml hygromycin and 15 μg/ml blasticidin. Cells were induced to express VCP/p97 by adding 3 μg/ml doxycycline to complete medium for 24 h.

### Transfections and virus stocks.

Small scale RNA transfections were performed by using *Trans*IT mRNA transfection reagent (Mirus Bio) according to manufacturer’s recommendations. BHK-NS1 stable cells were regenerated as previously described (23). Briefly, 1 μg of pSINrep21/YFV NS1 plasmid DNA was transfected into BHK-21 clone 15 cells by using 8 μl of *Trans*IT LT1 reagent (Mirus Bio, Wisconsin, MD) and low-serum Opti-MEM (Life Technologies). Transfected cells were then selected for 1 week in complete growth medium supplemented with 5 μg/ml puromycin. Reporter virus RNAs were transcribed from XhoI-linearized plasmid templates pYF17D/Nluc and pYFΔSK/Nluc by using SP6 RNA polymerase in the presence of the ARCA synthetic cap analog (New England Biolabs, Ipswich, MA). Primary stocks of reporter virus were generated by electroporation of BHK-21 clone 15 or BHK-NS1 cells with YFV RNA transcripts, as previously described (23). At 36 h postelectroporation, conditioned media containing primary YFV-17D/Nluc or YFVΔSK/Nluc stocks were harvested and clarified by centrifugation at 3,000 × *g* at 4°C for 10 min to remove cell debris.

Pilot experiments showed that primary stocks of YFV-17D/Nluc virus harvested at late time points (≥36 h posttransfection) contained high levels of Nluc released into the conditioned cell culture media, which correlated with the onset of virus-induced cytopathic effects. Therefore, to help minimize background, primary stocks of Nluc-expressing virus were dialyzed via ultrafiltration with Centricon plus-80 (containing 100-kDa nominal molecular weight cutoff polyethersulfone filters) to remove contaminating background Nluc activity. Furthermore, secondary virus stocks with minimal Nluc background were harvested at early (<24 h) times postinfection, albeit with reduced titers. Specifically, primary virus stocks were passaged on several 15-cm dishes of ∼70% confluent monolayers of BHK-21 or BHK-NS1 cells; after 1 h of incubation at 37°C, the inoculum was removed, washed twice with complete DMEM and twice with phosphate-buffered saline (PBS), and then incubated in the presence of complete DMEM containing 2% FBS. Conditioned cell culture media were harvested ∼18 h postinoculation, clarified as before, and stored in 1-ml aliquots at –80°C. These early harvest virus stocks had very low contaminating Nluc activity that was effectively removed by washing infected cells three times with PBS.

### Virus infectivity.

Infectivity measurements were determined by using plaque assays or endpoint dilution assays. Plaque assays were performed as previously described (90). For endpoint dilution assays with Nluc viruses, virus stocks were serially diluted in half-log (√10) intervals in DMEM containing 2% FBS and nonessential amino acids; each dilution was then added to BHK cells seeded in 96-well plates. At 24 h postinfection, cells were washed twice with complete DMEM and once with PBS, and the Nluc activity was measured in all wells. Wells were scored positive if the Nluc activity was >2σ from the mean of uninfected controls. Tissue culture infectious dose 50% endpoint (TCID_50_) values were calculated by using the method of Reed and Muench method, as previously described (58).

### Nluc activity.

Nluc activity was measured by using the Nano-Glo luciferase assay (Promega). At the indicated times in the assay, cells grown in 96-well plates were gently washed twice with complete DMEM and once with PBS and then lysed with 20 μl of Nano-Glo luciferase assay buffer containing the substrate. The Nluc activity was measured from cell lysates within 10 min of lysis (or substrate addition) by transferring lysate into white OptiPlate 96-well plates (Perkin-Elmer, Waltham, MA) and measured in a Berthold Centro XS3 LB 960 luminescent plate reader with readings integrated over 0.2 s.

### Inhibitors and treatments.

Ammonium chloride, BafA1, cycloheximide, and DBeQ were purchased from Millipore-Sigma (Burlington, MA). Pitstop2 and Dynasore were purchased from Abcam, Cambridge, MA. Pyr-41 was purchased from MedChemExpress (Monmouth Junction, NJ). NMS-873 was purchased from Tocris (Minneapolis, MN). Except where noted, all drugs were added to cells 1 h prior to infection or RNA transfection at the following concentrations: Pyr-41 (50 μM), DBeQ (10 μM), NMS-873 (300 nM), and CHX (100 μg/ml) and maintained in the cell culture medium throughout the experiments. The DENV fusion inhibitor K784-9103 was purchased from ChemDiv (San Diego, CA), and its structure and purity were confirmed by tandem liquid chromatography-mass spectrometry. Inhibition of E-mediated fusion was performed as previously described (31). Briefly, reporter viruses were incubated in medium containing the indicated concentrations of K784-9103 and then mixed in a rotary shaker for 30 min at room temperature to allow inhibitor to bind to virus particles before adding to cells.

DBeQ washout experiments were performed by adding 0.1% dimethyl sulfoxide (DMSO), 10 μM DBeQ, or 50 nM BafA1 with YFVΔSK/Nluc virus (i.e., no preincubation of cells). At 1 h postinfection, all cells were washed twice with complete medium and twice with PBS and then returned to media containing 0.1% (vol/vol) DMSO, 10 μM DBeQ, or 50 nM BafA1, as indicated. Samples were collected 6 h later.

RPLP1 was transiently knocked down in HeLa cells by reverse transfection of ON-TARGETplus Human RPLP1 SMARTPool small interfering RNA (siRNA; Dharmacon catalog no. L-011135-00-0005) or AllStars Negative Control siRNA (Qiagen catalog no. 1027280) as a negative control. Briefly, siRNA transfection mixtures were assembled with Lipofectamine RNAiMAX (Invitrogen) and plated in 96-well plates before seeding HeLa cells. After 48 h postseeding, the cells were used for infection experiments.

### Virus neutralization assay.

C57BL/6J IFNAR^−/−^ mice (91) were kindly provided by Sergei Kotenko, Rutgers University. Mice were bred in the Laboratory Animal Resource Center of Princeton University. All animal experiments were performed in accordance to protocol number 1930, which was reviewed and approved by the Institutional Animal Care and Use Committee (IACUC) of Princeton University. One female, 6-month-old mouse was infected intravenously with 1 × 10^7^ PFU of YFV-17D. At 18 days postinfection, the mouse was boosted intravenously with 1 × 10^7^ PFU of YFV-17D. Serum was collected 7 days after this booster injection. Serum from an uninfected female C57BL/6J IFNAR^−/−^ littermate (6 months old) was collected in parallel for use as a negative control. The YFV-immune human serum was obtained from a deidentified donor through the American Red Cross. Pooled human sera was purchased from Thermo-Fisher (Waltham, MA) for use as a negative control. For virus neutralization experiments, mouse and human sera were diluted into reporter virus stocks, and samples were incubated for 30 min at room temperature with tumbling. At the end of the incubation, samples were centrifuged briefly and 50-μl samples were added to three wells of BHK cells grown in 96-well plates. The Nluc expression was measured after 5 h of infection.

### Cytotoxicity assay.

Cells were seeded in 96-well plates and incubated with inhibitors at their respective experimental concentrations (indicated above) along with the membrane-impermeable nuclear dye Cytotox Green (Essen BioScience, Inc. Ann Arbor, MI) and cell-permeable Hoechst stain (Sigma). Cytotoxicity was measured on an ImageXpress Pico (Molecular Devices) by quantifying the fraction of Cytotox Green-positive, permeable cells among total cells.

### Immunostaining and FACS analysis.

WNV_KUN_ and ZIKV NS1 were detected by immunostaining with 6B8-2D8, a flavivirus NS1 cross-reactive monoclonal antibody, originally raised against DENV-4 NS1 (kindly provided by Marie Flamand, Institut Pasteur). Briefly, cells were washed twice with PBS and treated with Accumax (Innovative Cell Technologies, Inc., San Diego, CA) to gently dissociate cells for fluorescence-activated cell sorting (FACS) analysis. Dissociated cells were directly fixed in paraformaldehyde solution (2% [wt/vol] final) for 30 min at room temperature. Fixed cells were permeabilized with 0.2% saponin for ∼30 min on a rotating chamber, followed by two washes with PBS. Cells were incubated overnight with a 1:3,000 dilution of NS1-specific antibody 6B8-2D8 in PBS containing 2% FBS, washed with PBS, and incubated with 1:1500 dilution of an anti-mouse secondary antibody conjugated to Alexa 680 fluorescent dye. The specificity of labeling was confirmed by parallel incubation with the dye-conjugated secondary antibody in the absence of primary antibody and by performing the complete staining procedure on uninfected cells. At the end of incubation, the cells were washed twice, resuspended in PBS, and subjected to FACS analysis to quantify the percentage of NS1-positive cells by using the far-red channel.

## References

[1] Simmonds P, Becher P, Bukh J, Gould EA, Meyers G, Monath T, Muerhoff S, Pletnev A, Rico-Hesse R, Smith DB, Stapleton JT, Ictv RC 2017 ICTV virus taxonomy profile: *Flaviviridae*. J Gen Virol 98:2–3. doi:10.1099/jgv.0.000672. 28218572 PMC5370391

[2] Kuhn RJ, Zhang W, Rossmann MG, Pletnev SV, Corver J, Lenches E, Jones CT, Mukhopadhyay S, Chipman PR, Strauss EG, Baker TS, Strauss JH 2002 Structure of dengue virus: implications for flavivirus organization, maturation, and fusion. Cell 108:717–725. doi:10.1016/s0092-8674(02)00660-8. 11893341 PMC4152842

[3] Lindenbach BD, Murray CL, Thiel HJ, Rice CM 2013 *Flaviviridae*, p 712–746. In Knipe DM, Howley PM (ed), Fields virology, 6th ed Lippincott/Williams & Wilkins, Philadelphia, PA.

[4] Therkelsen MD, Klose T, Vago F, Jiang W, Rossmann MG, Kuhn RJ 2018 Flaviviruses have imperfect icosahedral symmetry. Proc Natl Acad Sci U S A 115:11608–11612. doi:10.1073/pnas.1809304115. 30348794 PMC6233072

[5] Tan TY, Fibriansah G, Kostyuchenko VA, Ng TS, Lim XX, Zhang S, Lim XN, Wang J, Shi J, Morais MC, Corti D, Lok SM 2020 Capsid protein structure in Zika virus reveals the flavivirus assembly process. Nat Commun 11:895. doi:10.1038/s41467-020-14647-9. 32060358 PMC7021721

[6] Lobigs M, Lee E 2004 Inefficient signalase cleavage promotes efficient nucleocapsid incorporation into budding flavivirus membranes. J Virol 78:178–186. doi:10.1128/jvi.78.1.178-186.2004. 14671099 PMC303399

[7] Lobigs M, Lee E, Ng ML, Pavy M, Lobigs P 2010 A flavivirus signal peptide balances the catalytic activity of two proteases and thereby facilitates virus morphogenesis. Virology 401:80–89. doi:10.1016/j.virol.2010.02.008. 20207389

[8] Amberg SM, Rice CM 1999 Mutagenesis of the NS2B-NS3-mediated cleavage site in the flavivirus capsid protein demonstrates a requirement for coordinated processing. J Virol 73:8083–8094. doi:10.1128/JVI.73.10.8083-8094.1999. 10482557 PMC112824

[9] Laureti M, Narayanan D, Rodriguez-Andres J, Fazakerley JK, Kedzierski L 2018 Flavivirus receptors: diversity, identity, and cell entry. Front Immunol 9:2180. doi:10.3389/fimmu.2018.02180. 30319635 PMC6168832

[10] Chu JJ, Ng ML 2004 Infectious entry of West Nile virus occurs through a clathrin-mediated endocytic pathway. J Virol 78:10543–10555. doi:10.1128/JVI.78.19.10543-10555.2004. 15367621 PMC516396

[11] Glasner DR, Puerta-Guardo H, Beatty PR, Harris E 2018 The good, the bad, and the shocking: the multiple roles of dengue virus nonstructural protein 1 in protection and pathogenesis. Annu Rev Virol 5:227–253. doi:10.1146/annurev-virology-101416-041848. 30044715 PMC6311996

[12] Luo D, Xu T, Hunke C, Gruber G, Vasudevan SG, Lescar J 2008 Crystal structure of the NS3 protease-helicase from dengue virus. J Virol 82:173–183. doi:10.1128/JVI.01788-07. 17942558 PMC2224403

[13] Issur M, Geiss BJ, Bougie I, Picard-Jean F, Despins S, Mayette J, Hobdey SE, Bisaillon M 2009 The flavivirus NS5 protein is a true RNA guanylyltransferase that catalyzes a two-step reaction to form the RNA cap structure. RNA 15:2340–2350. doi:10.1261/rna.1609709. 19850911 PMC2779676

[14] Steffens S, Thiel HJ, Behrens SE 1999 The RNA-dependent RNA polymerases of different members of the family *Flaviviridae* exhibit similar properties *in vitro*. J Gen Virol 80:2583–2590. doi:10.1099/0022-1317-80-10-2583. 10573150

[15] Koschinski A, Wengler G, Wengler G, Repp H 2003 The membrane proteins of flaviviruses form ion-permeable pores in the target membrane after fusion: identification of the pores and analysis of their possible role in virus infection. J Gen Virol 84:1711–1721. doi:10.1099/vir.0.19062-0. 12810864

[16] Kiermayr S, Kofler RM, Mandl CW, Messner P, Heinz FX 2004 Isolation of capsid protein dimers from the tick-borne encephalitis flavivirus and *in vitro* assembly of capsid-like particles. J Virol 78:8078–8084. doi:10.1128/JVI.78.15.8078-8084.2004. 15254179 PMC446133

[17] Byk LA, Iglesias NG, De Maio FA, Gebhard LG, Rossi M, Gamarnik AV 2016 Dengue virus genome uncoating requires ubiquitination. mBio 7:e00804-16. doi:10.1128/mBio.00804-16. 27353759 PMC4937216

[18] van den Boom J, Meyer H 2018 VCP/p97-mediated unfolding as a principle in protein homeostasis and signaling. Mol Cell 69:182–194. doi:10.1016/j.molcel.2017.10.028. 29153394

[19] Ye Y, Tang WK, Zhang T, Xia D 2017 A mighty “protein extractor” of the cell: structure and function of the p97/CDC48 ATPase. Front Mol Biosci 4:39. doi:10.3389/fmolb.2017.00039. 28660197 PMC5468458

[20] Davies JM, Brunger AT, Weis WI 2008 Improved structures of full-length p97, an AAA ATPase: implications for mechanisms of nucleotide-dependent conformational change. Structure 16:715–726. doi:10.1016/j.str.2008.02.010. 18462676

[21] Banerjee S, Bartesaghi A, Merk A, Rao P, Bulfer SL, Yan Y, Green N, Mroczkowski B, Neitz RJ, Wipf P, Falconieri V, Deshaies RJ, Milne JL, Huryn D, Arkin M, Subramaniam S 2016 2.3 Å resolution cryo-EM structure of human p97 and mechanism of allosteric inhibition. Science 351:871–875. doi:10.1126/science.aad7974. 26822609 PMC6946184

[22] Chapman E, Maksim N, de la Cruz F, La Clair JJ 2015 Inhibitors of the AAA+ chaperone p97. Molecules 20:3027–3049. doi:10.3390/molecules20023027. 25685910 PMC4576884

[23] Lindenbach BD, Rice CM 1997 *trans*-Complementation of yellow fever virus NS1 reveals a role in early RNA replication. J Virol 71:9608–9617. doi:10.1128/JVI.71.12.9608-9617.1997. 9371625 PMC230269

[24] Stoyanov CT, Boscardin SB, Deroubaix S, Barba-Spaeth G, Franco D, Nussenzweig RS, Nussenzweig M, Rice CM 2010 Immunogenicity and protective efficacy of a recombinant yellow fever vaccine against the murine malarial parasite *Plasmodium yoelii*. Vaccine 28:4644–4652. doi:10.1016/j.vaccine.2010.04.071. 20451637 PMC2935264

[25] Yi Z, Sperzel L, Nurnberger C, Bredenbeek PJ, Lubick KJ, Best SM, Stoyanov CT, Law LM, Yuan Z, Rice CM, MacDonald MR 2011 Identification and characterization of the host protein DNAJC14 as a broadly active flavivirus replication modulator. PLoS Pathog 7:e1001255. doi:10.1371/journal.ppat.1001255. 21249176 PMC3020928

[26] Schoggins JW, Dorner M, Feulner M, Imanaka N, Murphy MY, Ploss A, Rice CM 2012 Dengue reporter viruses reveal viral dynamics in interferon receptor-deficient mice and sensitivity to interferon effectors *in vitro*. Proc Natl Acad Sci U S A 109:14610–14615. doi:10.1073/pnas.1212379109. 22908290 PMC3437900

[27] England CG, Ehlerding EB, Cai W 2016 NanoLuc: a small luciferase is brightening up the field of bioluminescence. Bioconjug Chem 27:1175–1187. doi:10.1021/acs.bioconjchem.6b00112. 27045664 PMC4871753

[28] Bredenbeek PJ, Kooi EA, Lindenbach B, Huijkman N, Rice CM, Spaan WJ 2003 A stable full-length yellow fever virus cDNA clone and the role of conserved RNA elements in flavivirus replication. J Gen Virol 84:1261–1268. doi:10.1099/vir.0.18860-0. 12692292

[29] Hahn CS, Hahn YS, Rice CM, Lee E, Dalgarno L, Strauss EG, Strauss JH 1987 Conserved elements in the 3′ untranslated region of flavivirus RNAs and potential cyclization sequences. J Mol Biol 198:33–41. doi:10.1016/0022-2836(87)90455-4. 2828633

[30] van der Schaar HM, Rust MJ, Chen C, van der Ende-Metselaar H, Wilschut J, Zhuang X, Smit JM 2008 Dissecting the cell entry pathway of dengue virus by single-particle tracking in living cells. PLoS Pathog 4:e1000244. doi:10.1371/journal.ppat.1000244. 19096510 PMC2592694

[31] Lian W, Jang J, Potisopon S, Li PC, Rahmeh A, Wang J, Kwiatkowski NP, Gray NS, Yang PL 2018 Discovery of immunologically inspired small molecules that target the viral envelope protein. ACS Infect Dis 4:1395–1406. doi:10.1021/acsinfecdis.8b00127. 30027735 PMC6392429

[32] Hackett BA, Cherry S 2018 Flavivirus internalization is regulated by a size-dependent endocytic pathway. Proc Natl Acad Sci U S A 115:4246–4251. doi:10.1073/pnas.1720032115. 29610346 PMC5910848

[33] Mar KB, Rinkenberger NR, Boys IN, Eitson JL, McDougal MB, Richardson RB, Schoggins JW 2018 LY6E mediates an evolutionarily conserved enhancement of virus infection by targeting a late entry step. Nat Commun 9:3603. doi:10.1038/s41467-018-06000-y. 30190477 PMC6127192

[34] Campos RK, Wong B, Xie X, Lu YF, Shi PY, Pompon J, Garcia-Blanco MA, Bradrick SS 2017 RPLP1 and RPLP2 are essential flavivirus host factors that promote early viral protein accumulation. J Virol 91:e01706-16. 27974556 10.1128/JVI.01706-16 PMC5286887

[35] Guo F, Wu S, Julander J, Ma J, Zhang X, Kulp J, Cuconati A, Block TM, Du Y, Guo JT, Chang J 2016 A novel benzodiazepine compound inhibits yellow fever virus infection by specifically targeting NS4B protein. J Virol 90:10774–10788. doi:10.1128/JVI.01253-16. 27654301 PMC5110185

[36] Yang Y, Kitagaki J, Dai RM, Tsai YC, Lorick KL, Ludwig RL, Pierre SA, Jensen JP, Davydov IV, Oberoi P, Li CC, Kenten JH, Beutler JA, Vousden KH, Weissman AM 2007 Inhibitors of ubiquitin-activating enzyme (E1), a new class of potential cancer therapeutics. Cancer Res 67:9472–9481. doi:10.1158/0008-5472.CAN-07-0568. 17909057

[37] Wojcik C, Yano M, DeMartino GN 2004 RNA interference of valosin-containing protein (VCP/p97) reveals multiple cellular roles linked to ubiquitin/proteasome-dependent proteolysis. J Cell Sci 117:281–292. doi:10.1242/jcs.00841. 14657277

[38] Chou TF, Brown SJ, Minond D, Nordin BE, Li K, Jones AC, Chase P, Porubsky PR, Stoltz BM, Schoenen FJ, Patricelli MP, Hodder P, Rosen H, Deshaies RJ 2011 Reversible inhibitor of p97, DBeQ, impairs both ubiquitin-dependent and autophagic protein clearance pathways. Proc Natl Acad Sci U S A 108:4834–4839. doi:10.1073/pnas.1015312108. 21383145 PMC3064330

[39] Magnaghi P, D’Alessio R, Valsasina B, Avanzi N, Rizzi S, Asa D, Gasparri F, Cozzi L, Cucchi U, Orrenius C, Polucci P, Ballinari D, Perrera C, Leone A, Cervi G, Casale E, Xiao Y, Wong C, Anderson DJ, Galvani A, Donati D, O’Brien T, Jackson PK, Isacchi A 2013 Covalent and allosteric inhibitors of the ATPase VCP/p97 induce cancer cell death. Nat Chem Biol 9:548–556. doi:10.1038/nchembio.1313. 23892893

[40] Weisz OA 2003 Acidification and protein traffic. Int Rev Cytol 226:259–319. doi:10.1016/s0074-7696(03)01005-2. 12921239

[41] Gollins SW, Porterfield JS 1986 The uncoating and infectivity of the flavivirus West Nile on interaction with cells: effects of pH and ammonium chloride. J Gen Virol 67:1941–1950. doi:10.1099/0022-1317-67-9-1941. 3746254

[42] Gollins SW, Porterfield JS 1985 Flavivirus infection enhancement in macrophages: an electron microscopic study of viral cellular entry. J Gen Virol 66:1969–1982. doi:10.1099/0022-1317-66-9-1969. 4031825

[43] Helenius A 2018 Virus entry: looking back and moving forward. J Mol Biol 430:1853–1862. doi:10.1016/j.jmb.2018.03.034. 29709571 PMC7094621

[44] Klasse PJ 2015 Molecular determinants of the ratio of inert to infectious virus particles. Prog Mol Biol Transl Sci 129:285–326. doi:10.1016/bs.pmbts.2014.10.012. 25595808 PMC4724431

[45] van der Schaar HM, Rust MJ, Waarts BL, van der Ende-Metselaar H, Kuhn RJ, Wilschut J, Zhuang X, Smit JM 2007 Characterization of the early events in dengue virus cell entry by biochemical assays and single-virus tracking. J Virol 81:12019–12028. doi:10.1128/JVI.00300-07. 17728239 PMC2168764

[46] Potokar M, Korva M, Jorgačevski J, Avšič-Županc T, Zorec R 2014 Tick-borne encephalitis virus infects rat astrocytes but does not affect their viability. PLoS One 9:e86219. doi:10.1371/journal.pone.0086219. 24465969 PMC3896472

[47] Pierson TC, Diamond MS 2012 Degrees of maturity: the complex structure and biology of flaviviruses. Curr Opin Virol 2:168–175. doi:10.1016/j.coviro.2012.02.011. 22445964 PMC3715965

[48] Gehrke R, Heinz FX, Davis NL, Mandl CW 2005 Heterologous gene expression by infectious and replicon vectors derived from tick-borne encephalitis virus and direct comparison of this flavivirus system with an alphavirus replicon. J Gen Virol 86:1045–1053. doi:10.1099/vir.0.80677-0. 15784898

[49] He Y, Liu P, Wang T, Wu Y, Lin X, Wang M, Jia R, Zhu D, Liu M, Zhao X, Yang Q, Wu Y, Zhang S, Liu Y, Zhang L, Yu Y, Pan L, Chen S, Cheng A 2019 Genetically stable reporter virus, subgenomic replicon and packaging system of duck Tembusu virus based on a reverse genetics system. Virology 533:86–92. doi:10.1016/j.virol.2019.05.003. 31136895

[50] Kassar TC, Magalhães T, S JVJ, Carvalho AGO, Silva ANMRDA, Queiroz SRA, Bertani GR, Gil LHVG 2017 Construction and characterization of a recombinant yellow fever virus stably expressing *Gaussia* luciferase. An Acad Bras Cienc 89:2119–2130. doi:10.1590/0001-3765201720160196. 28746549

[51] Pierson TC, Diamond MS, Ahmed AA, Valentine LE, Davis CW, Samuel MA, Hanna SL, Puffer BA, Doms RW 2005 An infectious West Nile virus that expresses a GFP reporter gene. Virology 334:28–40. doi:10.1016/j.virol.2005.01.021. 15749120

[52] Puig-Basagoiti F, Deas TS, Ren P, Tilgner M, Ferguson DM, Shi PY 2005 High-throughput assays using a luciferase-expressing replicon, virus-like particles, and full-length virus for West Nile virus drug discovery. Antimicrob Agents Chemother 49:4980–4988. doi:10.1128/AAC.49.12.4980-4988.2005. 16304161 PMC1315944

[53] Tamura T, Fukuhara T, Uchida T, Ono C, Mori H, Sato A, Fauzyah Y, Okamoto T, Kurosu T, Setoh YX, Imamura M, Tautz N, Sakoda Y, Khromykh AA, Chayama K, Matsuura Y 2018 Characterization of recombinant *Flaviviridae* viruses possessing a small reporter tag. J Virol 92:e01582-17. doi:10.1128/JVI.01582-17. 29093094 PMC5752933

[54] Zou G, Xu HY, Qing M, Wang QY, Shi PY 2011 Development and characterization of a stable luciferase dengue virus for high-throughput screening. Antiviral Res 91:11–19. doi:10.1016/j.antiviral.2011.05.001. 21575658

[55] Zhang Q, Li N, Deng C, Zhang Z, Li X, Yoshii K, Ye H, Zhang B 2019 *trans*-Complementation of replication-defective Omsk hemorrhagic fever virus for antiviral study. Virol Sin doi:10.1007/s12250-019-00109-0. PMC6687815 30949960

[56] Hall MP, Unch J, Binkowski BF, Valley MP, Butler BL, Wood MG, Otto P, Zimmerman K, Vidugiris G, Machleidt T, Robers MB, Benink HA, Eggers CT, Slater MR, Meisenheimer PL, Klaubert DH, Fan F, Encell LP, Wood KV 2012 Engineered luciferase reporter from a deep sea shrimp utilizing a novel imidazopyrazinone substrate. ACS Chem Biol 7:1848–1857. doi:10.1021/cb3002478. 22894855 PMC3501149

[57] Khromykh AA, Sedlak PL, Guyatt KJ, Hall RA, Westaway EG 1999 Efficient trans-complementation of the flavivirus Kunjin NS5 protein but not of the NS1 protein requires its coexpression with other components of the viral replicase. J Virol 73:10272–10280. doi:10.1128/JVI.73.12.10272-10280.1999. 10559344 PMC113081

[58] Lindenbach BD 2009 Measuring HCV infectivity produced in cell culture and *in vivo*. Methods Mol Biol 510:329–336. doi:10.1007/978-1-59745-394-3_24. 19009272

[59] Płaszczyca A, Scaturro P, Neufeldt CJ, Cortese M, Cerikan B, Ferla S, Brancale A, Pichlmair A, Bartenschlager R 2019 A novel interaction between dengue virus nonstructural protein 1 and the NS4A-2K-4B precursor is required for viral RNA replication but not for formation of the membranous replication organelle. PLoS Pathog 15:e1007736. doi:10.1371/journal.ppat.1007736. 31071189 PMC6508626

[60] Youn S, Li T, McCune BT, Edeling MA, Fremont DH, Cristea IM, Diamond MS 2012 Evidence for a genetic and physical interaction between nonstructural proteins NS1 and NS4B that modulates replication of West Nile virus. J Virol 86:7360–7371. doi:10.1128/JVI.00157-12. 22553322 PMC3416313

[61] Akey DL, Brown WC, Dutta S, Konwerski J, Jose J, Jurkiw TJ, DelProposto J, Ogata CM, Skiniotis G, Kuhn RJ, Smith JL 2014 Flavivirus NS1 structures reveal surfaces for associations with membranes and the immune system. Science 343:881–885. doi:10.1126/science.1247749. 24505133 PMC4263348

[62] Lindenbach BD, Rice CM 1999 Genetic interaction of flavivirus nonstructural proteins NS1 and NS4A as a determinant of replicase function. J Virol 73:4611–4621. doi:10.1128/JVI.73.6.4611-4621.1999. 10233920 PMC112502

[63] Youn S, Ambrose RL, Mackenzie JM, Diamond MS 2013 Nonstructural protein-1 is required for West Nile virus replication complex formation and viral RNA synthesis. Virol J 10:339. doi:10.1186/1743-422X-10-339. 24245822 PMC3842638

[64] Zhang HL, Ye HQ, Deng CL, Liu SQ, Shi PY, Qin CF, Yuan ZM, Zhang B 2017 Generation and characterization of West Nile pseudo-infectious reporter virus for antiviral screening. Antiviral Res 141:38–47. doi:10.1016/j.antiviral.2017.02.006. 28202375

[65] Acosta EG, Castilla V, Damonte EB 2009 Alternative infectious entry pathways for dengue virus serotypes into mammalian cells. Cell Microbiol 11:1533–1549. doi:10.1111/j.1462-5822.2009.01345.x. 19523154 PMC7162254

[66] Krishnan MN, Sukumaran B, Pal U, Agaisse H, Murray JL, Hodge TW, Fikrig E 2007 Rab 5 is required for the cellular entry of dengue and West Nile viruses. J Virol 81:4881–4885. doi:10.1128/JVI.02210-06. 17301152 PMC1900185

[67] Mosso C, Galvan-Mendoza IJ, Ludert JE, del Angel RM 2008 Endocytic pathway followed by dengue virus to infect the mosquito cell line C6/36 HT. Virology 378:193–199. doi:10.1016/j.virol.2008.05.012. 18571214

[68] Acosta EG, Castilla V, Damonte EB 2008 Functional entry of dengue virus into Aedes albopictus mosquito cells is dependent on clathrin-mediated endocytosis. J Gen Virol 89:474–484. doi:10.1099/vir.0.83357-0. 18198378

[69] Heinz FX, Auer G, Stiasny K, Holzmann H, Mandl C, Guirakhoo F, Kunz C 1994 The interactions of the flavivirus envelope proteins: implications for virus entry and release. Arch Virol Suppl 9:339–348. doi:10.1007/978-3-7091-9326-6_34. 7913359

[70] Krishnan MN, Ng A, Sukumaran B, Gilfoy FD, Uchil PD, Sultana H, Brass AL, Adametz R, Tsui M, Qian F, Montgomery RR, Lev S, Mason PW, Koski RA, Elledge SJ, Xavier RJ, Agaisse H, Fikrig E 2008 RNA interference screen for human genes associated with West Nile virus infection. Nature 455:242–245. doi:10.1038/nature07207. 18690214 PMC3136529

[71] Fernandez-Garcia MD, Meertens L, Bonazzi M, Cossart P, Arenzana-Seisdedos F, Amara A 2011 Appraising the roles of CBLL1 and the ubiquitin/proteasome system for flavivirus entry and replication. J Virol 85:2980–2989. doi:10.1128/JVI.02483-10. 21191016 PMC3067953

[72] Wang S, Liu H, Zu X, Liu Y, Chen L, Zhu X, Zhang L, Zhou Z, Xiao G, Wang W 2016 The ubiquitin-proteasome system is essential for the productive entry of Japanese encephalitis virus. Virology 498:116–127. doi:10.1016/j.virol.2016.08.013. 27567260

[73] Greene W, Zhang W, He M, Witt C, Ye F, Gao SJ 2012 The ubiquitin/proteasome system mediates entry and endosomal trafficking of Kaposi’s sarcoma-associated herpesvirus in endothelial cells. PLoS Pathog 8:e1002703. doi:10.1371/journal.ppat.1002703. 22615563 PMC3355089

[74] Park CW, Ryu KY 2014 Cellular ubiquitin pool dynamics and homeostasis. BMB Rep 47:475–482. doi:10.5483/bmbrep.2014.47.9.128. 24924398 PMC4206721

[75] Xu Q, Farah M, Webster JM, Wojcikiewicz RJ 2004 Bortezomib rapidly suppresses ubiquitin thiolesterification to ubiquitin-conjugating enzymes and inhibits ubiquitination of histones and type I inositol 1,4,5-trisphosphate receptor. Mol Cancer Ther 3:1263–1269. 15486193

[76] McDowell GS, Philpott A 2013 Non-canonical ubiquitylation: mechanisms and consequences. Int J Biochem Cell Biol 45:1833–1842. doi:10.1016/j.biocel.2013.05.026. 23732108

[77] Li JM, Wu H, Zhang W, Blackburn MR, Jin J 2014 The p97-UFD1L-NPL4 protein complex mediates cytokine-induced IκBα proteolysis. Mol Cell Biol 34:335–347. doi:10.1128/MCB.01190-13. 24248593 PMC3911508

[78] Stolz A, Hilt W, Buchberger A, Wolf DH 2011 Cdc48: a power machine in protein degradation. Trends Biochem Sci 36:515–523. doi:10.1016/j.tibs.2011.06.001. 21741246

[79] Brandman O, Stewart-Ornstein J, Wong D, Larson A, Williams CC, Li GW, Zhou S, King D, Shen PS, Weibezahn J, Dunn JG, Rouskin S, Inada T, Frost A, Weissman JS 2012 A ribosome-bound quality control complex triggers degradation of nascent peptides and signals translation stress. Cell 151:1042–1054. doi:10.1016/j.cell.2012.10.044. 23178123 PMC3534965

[80] Verma R, Oania RS, Kolawa NJ, Deshaies RJ 2013 Cdc48/p97 promotes degradation of aberrant nascent polypeptides bound to the ribosome. Elife 2:e00308. doi:10.7554/eLife.00308. 23358411 PMC3552423

[81] Kloppsteck P, Ewens CA, Forster A, Zhang X, Freemont PS 2012 Regulation of p97 in the ubiquitin-proteasome system by the UBX protein-family. Biochim Biophys Acta 1823:125–129. doi:10.1016/j.bbamcr.2011.09.006. 21963883

[82] Hanzelmann P, Schindelin H 2017 The interplay of cofactor interactions and posttranslational modifications in the regulation of the AAA+ ATPase p97. Front Mol Biosci 4:21. doi:10.3389/fmolb.2017.00021. 28451587 PMC5389986

[83] Phongphaew W, Kobayashi S, Sasaki M, Carr M, Hall WW, Orba Y, Sawa H 2017 Valosin-containing protein (VCP/p97) plays a role in the replication of West Nile virus. Virus Res 228:114–123. doi:10.1016/j.virusres.2016.11.029. 27914931 PMC7114552

[84] Wong HH, Kumar P, Tay FP, Moreau D, Liu DX, Bard F 2015 Genome-wide screen reveals valosin-containing protein requirement for coronavirus exit from endosomes. J Virol 89:11116–11128. doi:10.1128/JVI.01360-15. 26311884 PMC4621105

[85] Panda D, Rose PP, Hanna SL, Gold B, Hopkins KC, Lyde RB, Marks MS, Cherry S 2013 Genome-wide RNAi screen identifies SEC61A and VCP as conserved regulators of Sindbis virus entry. Cell Rep 5:1737–1748. doi:10.1016/j.celrep.2013.11.028. 24332855 PMC3920290

[86] Carissimo G, Chan YH, Utt A, Chua TK, Bakar FA, Merits A, Ng L 2019 VCP/p97 is a proviral host factor for replication of chikungunya virus and other alphaviruses. Front Microbiol 10:2236. doi:10.3389/fmicb.2019.02236. 31636613 PMC6787436

[87] Yi Z, Fang C, Zou J, Xu J, Song W, Du X, Pan T, Lu H, Yuan Z 2016 Affinity purification of the hepatitis C virus replicase identifies valosin-containing protein, a member of the ATPases associated with diverse cellular activities family, as an active virus replication modulator. J Virol 90:9953–9966. doi:10.1128/JVI.01140-16. 27558430 PMC5068543

[88] Yi Z, Yuan Z 2017 Aggregation of a hepatitis C virus replicase module induced by ablation of p97/VCP. J Gen Virol 98:1667–1678. doi:10.1099/jgv.0.000828. 28691899

[89] Ernst R, Mueller B, Ploegh HL, Schlieker C 2009 The otubain YOD1 is a deubiquitinating enzyme that associates with p97 to facilitate protein dislocation from the ER. Mol Cell 36:28–38. doi:10.1016/j.molcel.2009.09.016. 19818707 PMC2774717

[90] Onorati M, Li Z, Liu F, Sousa AMM, Nakagawa N, Li M, Dell’Anno MT, Gulden FO, Pochareddy S, Tebbenkamp ATN, Han W, Pletikos M, Gao T, Zhu Y, Bichsel C, Varela L, Szigeti-Buck K, Lisgo S, Zhang Y, Testen A, Gao X-B, Mlakar J, Popovic M, Flamand M, Strittmatter SM, Kaczmarek LK, Anton ES, Horvath TL, Lindenbach BD, Sestan N 2016 Zika virus disrupts phospho-TBK1 localization and mitosis in human neuroepithelial stem cells and radial glia. Cell Rep 16:2576–2592. doi:10.1016/j.celrep.2016.08.038. 27568284 PMC5135012

[91] Lin JD, Feng N, Sen A, Balan M, Tseng HC, McElrath C, Smirnov SV, Peng J, Yasukawa LL, Durbin RK, Durbin JE, Greenberg HB, Kotenko SV 2016 Distinct roles of type I and type III interferons in intestinal immunity to homologous and heterologous rotavirus infections. PLoS Pathog 12:e1005600. doi:10.1371/journal.ppat.1005600. 27128797 PMC4851417

