# Supplementary material for: A Sensitive Yellow Fever Virus Entry Reporter Identifies Valosin-Containing Protein (VCP/p97) as an Essential Host Factor for Flavivirus Uncoating
Source: mBio. 2020 Apr 14;11(2):e00467-20. doi: 10.1128/mBio.00467-20 (PMC7157815; doi:10.1128/mBio.00467-20)
Supplement: FIG S1 [file mBio.00467-20-sf001.pdf]

Fig.S1 Sequence alignment of human TERA(VCP/p97)with Aedes TER94
A.Input Sequences

>sp|P55072|TERA_HUMAN Transitional endoplasmic reticulum ATPase OS=Homo sapiens OX=9606 GN=VCP PE=1 SV=4
MASGADSKGDDLSTAILKQKNRPNRLIVDEAINEDNSVVSLSQPKMDELQLFRGDTVLLKGKKRREAVC
IVLSDDTCSDEKIRMNRVVRNNLRVRLGDVISIQPCPDVKYGKRIHVLPIDDTVEGITGNLFEVYLKPY
FLEAYRPIRKGDIFLVRGGMRAVEFKVVETDPSPYCIVAPDTVIHCEGEPIKREDEEESLNEVGYDDIG
GCRKQLAQIKEMVELPLRHPALFKAIGVKPPRGILLYGPPGTGKTLIARAVANETGAFFFLINGPEIMS
KLAGESESNLRKAFEEAEKNAPAIIFIDELDAIAPKREKTHGEVERRIVSQLLTLMDGLKQRAHVIVMA
ATNRPNSIDPALRRFGRFDREVDIGIPDATGRLEILQIHTKNMKLADDVDLEQVANETHGHVGADLAAL
CSEAALQAIRKKMDLIDLEDETIDAEVMNSLAVTMDDFRWALSQSNPSALRETVVEVPQVTWEDIGGLE
DVKRELQELVQYPVEHPDKFLKFGMTPSKGVLFYGPPGCGKTLLAKAIANECQANFISIKGPELLTMWF
GESEANVREIFDKARQAAPCVLFFDELDSIAKARGGNIGDGGGAADRVINQILTEMDGMSTKKNVFIIG
ATNRPDIIDPAILRPGRLDQLIYIPLPDEKSRVAILKANLRKSPVAKDVDLEFLAKMTNGFSGADLTEI
CQRACKLAIRESIESEIRRERERQTNPSAMEVEEDDPVPEIRRDHFEEAMRFARRSVSDNDIRKYEMFA
QTLQQSRGFGSFRFPSGNQGGAGPSQGSGGGTGGSVYTEDNDDDLYG

>XP_001654680.1 transitional endoplasmic reticulum ATPase TER94 [Aedes aegypti]
MAEGKNEDLATAILKRKERPNRLIVDEAGNDDNSVISLSQAKMDELQLFRGDTVLLKGKRRKETVCIVLS
DDNCPDEKIRMNRVVRNNLRVRLGDVVSIQSCPDVKYGKRVHILPIDDTVEGLTGNLFDVYLRPYFLEAY
RPIHSDDTFIVRGGMRAVEFKVVGADPSPYCIVAPETVIHCEGDPIKREEEEEEALNAVGYDDIGGCRKQL
AQIKEMVELPLRHPSLFKAIGVKPPRGILMYGPPGTGKTLIARAVANETGAFFFLINGPEIMSKLAGESE
SNLRKAFEEAEKKSPAIIFIDELDAIAPKREKTHGEVERRIVSQLLTLMDGMKKSSHVIVMAATNRPNSI
DPALRRFGRFDREIDIGIPDATGRLEVLRIHSKNMKLADDVDLEQIAAESHGHVGADLASLCSEAALQQI
REKMDLIDLEDDQIDAEVLNSLAVSMENFRYAMTKSSPSALRETVVEVPNTTWHDIGGLENVKRELQELV
QYPVEHPDKFLKFGMQPSRGVLFYGPPGCGKTLLAKAIANECQANFISVKGPELLTMWFGESEANVRDIF
DKARSASPCVLFFDELDSIAKSRGGNVGDAGGAADRVINQILTEMDGMGAKKNVFIIGATNRPDIIDPAI
LRPGRLDQLIYIPLPDDKSREAILKANLRKSPVAGDVDLTYVAKVTQGFSGADLTEICQRACKLAIRQAI
EAEIRRERERTEGQSSAMDMDEDDPVPNITRAHFEEAMKFARRSVSDNDIRKYEMFAQTLQQSRGFGSNF
RFPGGQSGSSSQGQGSSQPTSNPADNGDDDLYS

>XP_019533679.1 transitional endoplasmic reticulum ATPase TER94 [Aedes albopictus]
MAEGKNEDLATAILKRKERPNRLIVDEAGNDDNSVISLSQAKMDELQLFRGDTVLLKGKRRKETVCIVLS
DDNCPDEKIRMNRVVRNNLRVRLGDVVSIQSCPDVKYGKRVHILPIDDTVEGLTGNLFDVYLRPYFLEAY
RPIHSDDTFIVRGGMRAVEFKVVGADPSPYCIVAPETVIHCEGDPIKREEEEEEALNAVGYDDIGGCRKQL
AQIKEMVELPLRHPSLFKAIGVKPPRGILMYGPPGTGKTLIARAVANETGAFFFLINGPEIMSKLAGESE
SNLRKAFEEAEKNSPAIIFIDELDAIAPKREKTHGEVERRIVSQLLTLMDGMKKSSHVIVMAATNRPNSI
DPALRRFGRFDREIDIGIPDATGRLEVLRIHSKNMKLADDVDLEQIAAESHGHVGADLASLCSEAALQQI
REKMDLIDLEDDQIDAEVLNSLAVSMENFRYAMTKSSPSALRETVVEVPNTTWHDIGGLENVKRELQELV
QYPVEHPDKFLKFGMQPSRGVLFYGPPGCGKTLLAKAIANECQANFISVKGPELLTMWFGESEANVRDIF
DKARSASPCVLFFDELDSIAKSRGGNVGDAGGAADRVINQILTEMDGMGAKKNVFIIGATNRPDIIDPAI
LRPGRLDQLIYIPLPDDKSREAILKANLRKSPVAGDVDLTYVAKVTQGFSGADLTEICQRACKLAIRQAI
EAEIRRERERTEGQSSAMDMDEDDPVPNITRAHFEEAMKFARRSVSDNDIRKYEMFAQTLQQSRGFGNNF
RFPGGQSGSSSQGQGSSQPTSNPADNGDDDLYS

B. Alignment

CLUSTAL multiple sequence alignment by MUSCLE (3.8)

- Green residues indicate active site residues (K251 and K524 in human VCP/p97)
- Yellow residues indicate allosteric inhibitor binding residues (K615 and N617 in human VCP/p97)

```
sp|P55072|TERA_HUMAN      MASGADSKGDDLSTAILKQKNRPNRLIVDEAINEDNSVVSLSQPKMDELQLFRGDTVLLK 60
XP_001654680.1            ---MAEGKNEDLATAILKRKERPNRLIVDEAGNDDNSVISLSQAKMDELQLFRGDTVLLK
XP_019533679.1            ---MAEGKNEDLATAILKRKERPNRLIVDEAGNDDNSVISLSQAKMDELQLFRGDTVLLK
                              *:.*.:**:*****.*:********** *:****:****.****************

sp|P55072|TERA_HUMAN      GKKRREAVCIVLSDDTCSDEKIRMNRVVRNNLRVRLGDVISIQPCPDVKYGKRIHVLPID 120
XP_001654680.1            GKRRKETVCIVLSDDNCPDEKIRMNRVVRNNLRVRLGDVVSIQSCPDVKYGKRVHILPID
XP_019533679.1            GKRRKETVCIVLSDDNCPDEKIRMNRVVRNNLRVRLGDVVSIQSCPDVKYGKRVHILPID
                          **.*.*:********.*.*********************:***.*********:*:****

sp|P55072|TERA_HUMAN      DTVEGITGNLFEVYLKPYFLEAYRPIRKGDIFLVRGGMRAVEFKVVETDPSPYCIVAPDT 180
XP_001654680.1            DTVEGLTGNLFDVYLRPYFLEAYRPIHSDDTFIVRGGMRAVEFKVVGADPSPYCIVAPET
XP_019533679.1            DTVEGLTGNLFDVYLRPYFLEAYRPIHSDDTFIVRGGMRAVEFKVVGADPSPYCIVAPET
                          *****:*****:***.**********...* *:************* :**********:*

sp|P55072|TERA_HUMAN      VIHCEGEPIKREDEEESLNEVGYDDIGGCRKQLAQIKEMVELPLRHPALFKAIGVKPPRG 240
XP_001654680.1            VIHCEGDPIKREEEEEALNAVGYDDIGGCRKQLAQIKEMVELPLRHPSLFKAIGVKPPRG
XP_019533679.1            VIHCEGDPIKREEEEEALNAVGYDDIGGCRKQLAQIKEMVELPLRHPSLFKAIGVKPPRG
                          ******:*****:***:** ***************************:************

sp|P55072|TERA_HUMAN      ILLYGPPGTGKTLIARAVANETGAFFFLINGPEIMSKLAGESESNLRKAFEEAEKNAPAI 300
XP_001654680.1            ILMYGPPGTGKTLIARAVANETGAFFFLINGPEIMSKLAGESESNLRKAFEEAEKKSPAI
XP_019533679.1            ILMYGPPGTGKTLIARAVANETGAFFFLINGPEIMSKLAGESESNLRKAFEEAEKNSPAI
                          **:****************************************************::***

sp|P55072|TERA_HUMAN      IFIDELDAIAPKREKTHGEVERRIVSQLLTLMDGLKQRAHVIVMAATNRPNSIDPALRRF 360
XP_001654680.1            IFIDELDAIAPKREKTHGEVERRIVSQLLTLMDGMKKSSHVIVMAATNRPNSIDPALRRF
XP_019533679.1            IFIDELDAIAPKREKTHGEVERRIVSQLLTLMDGMKKSSHVIVMAATNRPNSIDPALRRF
                          **********************************:*: :*********************

sp|P55072|TERA_HUMAN      GRFDREVDIGIPDATGRLEILQIHTKNMKLADDVDLEQVANETHGHVGADLAALCSEAAL 420
XP_001654680.1            GRFDREIDIGIPDATGRLEVLRIHSKNMKLADDVDLEQIAAESHGHVGADLASLCSEAAL
XP_019533679.1            GRFDREIDIGIPDATGRLEVLRIHSKNMKLADDVDLEQIAAESHGHVGADLASLCSEAAL
                          ******:************:*.**:*************:* *:*********:*******

sp|P55072|TERA_HUMAN      QAIRKKMDLIDLEDETIDAEVMNSLAVTMDDFRWALSQSNPSALRETVVEVPQVTWEDIG 480
XP_001654680.1            QQIREKMDLIDLEDDQIDAEVLNSLAVSMENFRYAMTKSSPSALRETVVEVPNTTWHDIG
XP_019533679.1            QQIREKMDLIDLEDDQIDAEVLNSLAVSMENFRYAMTKSSPSALRETVVEVPNTTWHDIG
                          * **:*********: *****:*****:*:::**:*:::*.************:.** ***
```

```
sp|P55072|TERA_HUMAN      GLEDVKRELQELVQYPVEHPDKFLKFGMTPSKGVLFYGPPGCGKTLLAKAIANECQANFI 540
XP_001654680.1            GLENVKRELQELVQYPVEHPDKFLKFGMQPSRGVLFYGPPGCGKTLLAKAIANECQANFI
XP_019533679.1            GLENVKRELQELVQYPVEHPDKFLKFGMQPSRGVLFYGPPGCGKTLLAKAIANECQANFI
                          ***:************************ **.****************************

sp|P55072|TERA_HUMAN      SIKGPELLTMWFGESEANVREIFDKARQAAPCVLFFDELDSIAKARGGNIGDGGGAADRV 600
XP_001654680.1            SVKGPELLTMWFGESEANVRDIFDKARSASPCVLFFDELDSIAKSRGGNVGDAGGAADRV
XP_019533679.1            SVKGPELLTMWFGESEANVRDIFDKARSASPCVLFFDELDSIAKSRGGNVGDAGGAADRV
                          *:******************:******.*:**************:****:**.*******

sp|P55072|TERA_HUMAN      INQILTEMDGMSTKKNVFIIGATNRPDIIDPAILRPGRLDQLIYIPLPDEKSRVAILKAN 660
XP_001654680.1            INQILTEMDGMGAKKNVFIIGATNRPDIIDPAILRPGRLDQLIYIPLPDDKSREAILKAN
XP_019533679.1            INQILTEMDGMGAKKNVFIIGATNRPDIIDPAILRPGRLDQLIYIPLPDDKSREAILKAN
                          ***********.:************************************:*** ******

sp|P55072|TERA_HUMAN      LRKSPVAKDVDLEFLAKMTNGFSGADLTEICQRACKLAIRESIESEIRRERER-QTNPSA 719
XP_001654680.1            LRKSPVAGDVDLTYVAKVTQGFSGADLTEICQRACKLAIRQAIEAEIRRERERTEGQSSA
XP_019533679.1            LRKSPVAGDVDLTYVAKVTQGFSGADLTEICQRACKLAIRQAIEAEIRRERERTEGQSSA
                          ******* **** ::**:*:********************::**:******** : :.**

sp|P55072|TERA_HUMAN      MEVEEDDPVPEIRRDHFEEAMRFARRSVSDNDIRKYEMFAQTLQQSRGFGS-FRFPSGNQ 778
XP_001654680.1            MDMDEDDPVPNITRAHFEEAMKFARRSVSDNDIRKYEMFAQTLQQSRGFGSNFRFPGGQS
XP_019533679.1            MDMDEDDPVPNITRAHFEEAMKFARRSVSDNDIRKYEMFAQTLQQSRGFGNNFRFPGGQS
                          *:::******:* * ******.****************************. ****.*:.

sp|P55072|TERA_HUMAN      GGAGPSQGSGGGTGGSVYTEDNDDDLYG 806
XP_001654680.1            GSSSQGQGSSQPTSNP--ADNGDDDLYS
XP_019533679.1            GSSSQGQGSSQPTSNP--ADNGDDDLYS
                          *.:. .***.  *...  :::.*****.
```

C.Identity Matrix

```
Percent Identity Matrix - created by Clustal2.1

     1: sp|P55072|TERA_HUMAN  100.00   84.89   84.89
     2: XP_001654680.1         84.89  100.00   99.75
     3: XP_019533679.1         84.89   99.75  100.00
```
